# Clinical use of neuro-imaging in psychiatric patients at the Charlotte Maxeke Johannesburg Academic Hospital

**DOI:** 10.4102/sajpsychiatry.v27i0.1614

**Published:** 2021-05-28

**Authors:** Bokang L. Letlotlo, Lavinia D. Lumu, Mahomed Y.H. Moosa, Fatima Y. Jeenah

**Affiliations:** 1 Department of Psychiatry, Faculty of Neurosciences, University of the Witwatersrand, Johannesburg, South Africa

**Keywords:** neuro-imaging, CT scans, psychiatric disorders, guidelines, a yield of abnormal scans, factors associated with abnormal scans, resource constraints, cost-effectiveness

## Abstract

**Background:**

Neuro-imaging is relatively new in psychiatry. Although the actual role of neuro-imaging in psychiatry remains unclear, it is used to strengthen clinical evidence in making psychiatric diagnoses.

**Aim:**

To analyse the records of inpatients referred for neuro-imaging (computerised tomography [CT] and/or magnetic resonance imaging [MRI] scans) to determine the proportion of abnormal neuro-imaging results and, if any, factors associated with abnormal neuro-imaging results.

**Setting:**

This study was conducted at the Charlotte Maxeke Johannesburg Academic Hospital (CMJAH) situated in Johannesburg, South Africa.

**Methods:**

This was a quantitative retrospective record review. All adult psychiatric inpatients who had undergone a CT and/or MRI scan during 01 January 2014 to 31 December 2015 were included. Out-patients or patients admitted in the medical wards were excluded from the study. All neuro-imaging referrals were identified from hospital records and their demographics, scan characteristics and diagnoses were subsequently captured.

**Results:**

A total of 1040 patients were admitted to the CMJAH psychiatric unit, of which 213 (20.5%) underwent neuro-imaging tests. Of the 213 scans performed, 74 were abnormal, representing a yield of 34.7%. The most common reported pathology was atrophy (*n* = 22, 29.7%). There was no statistically significant association between age group (*χ*^2^ = 3.9, *p* = 0.8), gender (*χ*^2^ = 1.3; *p* = 0.5), psychiatric diagnoses and abnormal scans. However, there were trends towards an association with comorbid HIV infection (*χ*^2^ = 3.476, *p* = 0.062) and comorbid substance abuse (*χ*^2^ = 2.286, *p* = 0.091).

**Conclusion:**

This study supports the need for clear clinical indications to justify the cost-effective use of neuro-imaging in psychiatry. This study’s high yield of abnormal CT scans, although similar to other studies, advocates that HIV positive testing and the presence of focal neurological signs will improve the yield further.

## Introduction

Neuro-imaging is relatively new and was first utilised as a tool in psychiatry in the 1980s.^1^ It may be divided into two main groups: structural imaging (which assesses the structural anatomy of the brain) and functional imaging (which assesses the physiological functioning of the brain).^2^ Functional neuro-imaging which assists the clinician to measure treatment response is recommended over structural neuro-imaging in psychiatry.^3^ Although the actual role of structural neuro-imaging in psychiatry remains unclear, it is used to strengthen the available clinical evidence in the confirmation of psychiatric diagnoses.^4,5^

First-episode psychosis, bipolar disorder, dementia and intellectual disability are some of the indications for neuro-imaging in the practice of psychiatry.^6^ However, routine neuro-imaging of first-episode psychoses in patients without focal neurological signs is not recommended as it has a low yield for pathology detection and is unlikely to significantly alter management.^5,7,8^ The use of neuro-imaging in bipolar disorder is indicated only when an organic aetiology is suspected.^6^ When a more sensitive neuro-imaging modality such as magnetic resonance imaging (MRI) is performed, the clinical significance of the results and incidental findings is not clear.^9,10^ In clinically suspected dementia, the correlation between the extent of atrophy on the scan and actual cognitive decline that needs to be determined.^11^ The use of neuro-imaging in intellectual disability is not routinely recommended. It is common for many medical illnesses to present with psychiatric symptoms; therefore, they are often referred to either medical or psychiatric specialties. Neuro-imaging has a role in the exclusion of medical conditions such as traumatic brain injuries, tumours, infections, infarctions and bleeding.^4,5,12,13,14,15^

The guidelines for neuro-imaging indications in psychiatry vary internationally and locally, with no clear overall consensus. Hollister and Shah^16^ recommend neuro-imaging in psychiatric patients for new or unexplained focal neurologic signs, for confirmation of a clinical diagnosis of Alzheimer’s disease and for first-episode psychosis (FEP) or personality change in patients over 50 years of age. The American Psychiatric Association posits that ‘[a] neuro-imaging finding to have diagnostic value must have sensitivity and specificity of no less than 80% verified by at least two independent studies, the psychiatric imaging literature does not support the application of neuro-imaging in psychiatric diagnostics or treatment and neuro-imaging has not had a significant impact on the diagnosis and treatment of psychiatric disorders’.^17^ The South African Society of Psychiatrists^6^ guidelines recommend neuro-imaging to assist with the management and not a diagnosis of psychiatric disorders: in first-episode psychotic patients who present with atypical symptoms or abnormal clinical findings, in dementia to clarify the aetiological diagnosis and in bipolar disorder with a suspected medical condition.

Charlotte Maxeke Johannesburg Academic Hospital (CMJAH) is an academic hospital, with 1018 approved inpatients’ beds, situated in Johannesburg, South Africa. The practice at CMJAH’s psychiatric unit is to conduct neuro-imaging as a baseline investigation for all admitted first presentations to psychiatry or when such instances were medically indicated. The Radiology Department is a 24-h service and the neuro-imaging modalities available include MRI, computerised tomography (CT) and single-photon emission computed tomography (SPECT) scans. Charlotte Maxeke Johannesburg Academic Hospital’s Radiology Department has an ‘emergency’ and ‘as soon as possible’ lists for the psychiatric inpatients. Emergency scans are done on the same day and the scans on the ‘as soon as possible’ list are done within 2 weeks of the request.

Neuro-imaging may be considered an expensive investigative tool in resource-limited health systems. To integrate neuro-imaging into routine use in psychiatric disorders, whilst simultaneously preventing overuse, there needs to be a convincing case wherein such investigations can lower the direct or indirect costs of these disorders.^4,18,19^ Hence, the need for this study is to determine the local yield and the clinical factors associated with abnormal scans. A better understanding of these factors may contribute to the review of the standard procedures relating to indications for neuro-imaging with a better yield of abnormalities, and that would be more cost-effective.

## Aim

This study aimed to analyse the records of a group of psychiatric inpatients referred for neuro-imaging (CT and/or MRI scans) at CMJAH during 01 January 2014 to 31 December 2015. The specific objectives were to determine the proportion of abnormal neuro-imaging results, and to determine, if any, associations between demographic, clinical and scan characteristics and abnormal neuro-imaging results.

## Method

This study was a quantitative retrospective record review. The population consisted of all adult (aged above 18 years) psychiatric inpatients who had CT and/or MRI scans conducted during the study period from 01 January 2014 to 31 December 2015. Patients were excluded if they were out-patients or patients admitted to medical wards.

The clinical records, kept in the CMJAH records filing system, were retrieved manually from the list of patients identified to have been admitted in the adult psychiatric ward during the study period. All the patients who were referred for CT and/or MRI scans were identified and their demographic, clinical and scan characteristics were captured on a data collecting sheet.

### Ethical considerations

Ethical approval to conduct the study was obtained from the Human Research and Ethics Committee of the University of the Witwatersrand (Study number: M150809). Confidentiality was maintained for each record by allocating a unique study number and no personal identifiers were used during capturing, analysis or discussion of data.

## Data analysis

Descriptive statistics were computed as counts and percentages, minimum and maximum and means (standard deviations – SD). Chi-square and Fischer’s tests were used to determine the relationship between general categorical characteristics (age groups, sex and clinical and laboratory findings) between the groups with abnormal and normal scans. A value of *p* < 0.05 was considered significant.

## Results

During the study period, 1040 adult patients were admitted to the CMJAH psychiatric unit. Of these patients, 213 (20.5%) underwent neuro-imaging scan.

The mean age of the study population was 38.8 years (SD = 15.41; range: 18–88). The mean age of the group with normal scans was 33.5 years (SD = 15.1; range: 18–82) and those with abnormal scans was 41.5 years (SD = 16.2; range: 19–88). There was no statistical association between mean age and abnormal scans (*χ*^2^ = 0.613, *p* = 0.894).

The majority of patients in the study population were in the 30–49 years age group (*n* = 91, 42.7%) and were males (*n* = 112, 52.6%). Approximately half (*n* = 95, 44.6%) of them underwent first admissions to psychiatry (Table 1).

**TABLE 1:** Frequency distribution of the demographic and clinical characteristics of the study population.

Variables	Total study population (*n* = 213)		Group with normal scans (*n* = 139)		Group with abnormal scans (*n* = 74)		Statistical comparisons between the two groups and variables
	*N*	%	*N*	%	*N*	%	
**Age group (years)**							
18–29	71	33.3	47	33.8	24	32.4	*χ*^2^ = 0.613, *p* = 0.893
30–49	91	42.7	61	43.9	30	40.5	-
50–59	20	9.4	12	8.6	8	10.8	-
> 60	31	14.5	19	13.7	12	16.3	-
**Gender**							
Female	100	46.9	62	44.6	38	51.4	*p* = 0.389
Male	112	52.6	76	54.7	36	48.6	-
**Psychiatric admissions**							
First	94	46.9	64	46.0	30	41.7	*p* = 0.647
Multiple	96	44.6	62	44.6	34	45.9	-
**Psychiatric diagnostic groups**							
Psychotic disorder	134	62.9	89	64.0	45	60.8	*χ*^2^ = 0.098, *p* = 0.756
Mood disorder	88	41.3	61	43.9	27	36.5	*χ*^2^ = 0.806, *p* = 0.369
Neurocognitive disorder	82	38.5	52	37.4	30	40.5	*χ*^2^ = 0.089, *p* = 0.765
Other (anxiety/personality/eating)	28	13.1	23	12.9	5	6.8	-
**Comorbid medical diagnosis**							
HIV	52	24.4	40	28.8	12	16.2	*χ*^2^ = 3.476, *p* = 0.062
Infections	41	19.2	26	18.7	15	20.3	*χ*^2^ = 0.009, *p* = 0.926
Neurological disorder	49	23.0	27	19.4	22	29.7	*χ*^2^ = 2.343, *p* = 0.126
Other (diabetes/dyslipidaemia)	13	6.1	8	5.8	5	6.8	-
**Comorbid substance use**							
Yes	66	31	49	35.3	17	23.0	*χ*^2^ = 2.855, *p* = 0.091
No	147	69	90	64.7	57	77.0	-

Of the total study population, 64.3% (*n* = 137) had more than one DSM-IV-TR/5 diagnosis. The most common psychiatric diagnoses were psychotic disorders (*n* = 134, 33%), followed by mood disorders (*n* = 88, 22%) and neurocognitive disorders (*n* = 82, 20%). Amongst the psychotic disorders group diagnosis, the majority were psychotic disorders because of a medical condition (*n* = 44, 32.5%) (Table 1).

Nearly three quarters of the patients (*n* = 150, 70.4%) had a comorbid medical illness, of which 32 (21.3%) had multiple medical diagnoses. The medical diagnoses included human immunodeficiency virus (HIV) (*n* = 52, 28.4%), neurological illnesses (*n* = 49, 26.8%), infections (*n* = 41, 22.4%), hypertension (*n* = 28, 15.3%), diabetes (*n* = 7, 3.8%) and dyslipidaemia (*n* = 6, 3.3%). Approximately one-third of the patients (*n* = 66, 31%) had a comorbid substance-related disorders (Table 1).

The most frequently performed neuro-imaging modality in the study population was CT brain imaging (*n* = 198, 93%), whilst five patients (2.3%) had MRI scan only and 10 (4.7%) had both forms of neuro-imaging performed. The urgency for the neuro-imaging scans was unknown in the majority of cases (*n* = 183, 85.9%), with only 8% (*n* = 17) considered as ‘emergency’. In the majority of cases, it was not known whether they had undergone a previous scan (*n* = 198, 93%) (Table 2).

**TABLE 2:** Frequency distribution of the scan characteristics of the study population.

Variables	Total study population (*n* = 213)		Group with normal scans (*n* = 139)		Group with abnormal scans (*n* = 74)		Statistical comparisons between the two groups and variables
	*N*	%	*N*	%	*N*	%	
**Type of scan**							
CT only	198	93.0	130	93.5	68	91.9	-
MRI only	5	2.3	4	2.8	1	1.4	-
Both CT and MRI	10	4.7	5	3.6	5	6.8	*χ*^2^ = 1.5, *p* = 0.5
**Urgency for scan**							
Elective	13	6.1	5	3.6	6	8.1	-
Emergency	17	8.0	8	5.8	9	12.2	*p* = 0.934 (Fisher’s)
**Frequency of scan**							
First	4	1.9	3	2.2	1	1.4	-
Repeat	11	5.2	5	3.6	6	8.1	*p* = 0.569 (Fisher’s)

CT, computerised tomography; MRI, magnetic resonance imaging.

Seventy-four of the 213 scans done were reported as abnormal, representing a yield of 34.7%. Seven of the abnormal scan reports by the radiologist were missing. The most common reported pathology by the radiologist was atrophy (*n* = 22, 29.7%).

### Socio-demographic and clinical characteristics associated with abnormal scans

The majority of abnormal scans were in the 30–39-year age group (*n* = 30, 40.5%), and showed an almost equal distribution amongst women and men (*n* = 38, 51.4% vs. *n* = 36, 48.6%). There were no statistically significant associations between age group (*χ*^2^ = 3.9, *p* = 0.8), gender (*χ*^2^ = 1.3; *p* = 0.5) and abnormal scans (Table 1).

The most common psychiatric diagnosis amongst those with abnormal scans was psychotic disorder (*n* = 45; 60.8%), followed by neurocognitive disorder (*n* = 30, 40.5%) and mood disorder (*n* = 27, 36.5%) (Table 2). There were no statistically significant associations between abnormal scans and any of the specific psychiatric diagnoses (*p* > 0.05) (Table 1).

The most common comorbid medical diagnosis amongst those with abnormal scans was a neurological condition (*n* = 22, 29.7%), followed by infections (*n* = 15, 20.3%) and HIV (*n* = 12, 16.2%) (Table 1). There was a trend towards an association with abnormal scans in those with HIV (*χ*^2^ = 2.343, *p* = 0.126, odds ratio [OR] = 0.569) compared to other medical conditions. There were no statistically significant associations with having a comorbid medical diagnosis of diabetes (*χ*^2^ = 0.1215, *p* = 0.737, OR = 1.343), infections (*χ*^2^ = 0.0087, *p* = 0.926, OR = 0.905); dyslipidaemia (*χ*^2^ = 0.131, *p* = 0.718) or neurological conditions (*χ*^2^ = 2.343, *p* = 0.126, OR = 0.569) (Table 1). Seventeen (23.0%) patients with abnormal scans had a comorbid substance use disorder.

Although not statistically significant, there was a trend towards an association between the presence of comorbid substance abuse and an abnormal CT scan (*χ*^2^ = 2.286, *p* = 0.091, OR = 1.825) (Table 1).

In approximately half of the group with normal (*n* = 64, 46%) and abnormal scans (*n* = 30, 41.7%), this was their first admission to psychiatry (*p* = 0.647) (Table 1). There were no significant associations between abnormal scans and the type of scan (*χ*^2^ = 1.5, *p* = 0.5), the urgency of the scan (*χ*^2^ = 3.7, *p* = 0.2) and the frequency of the scan (*χ*^2^ = 2.1, *p* = 0.3) (Table 2).

## Discussion

This study found that approximately one-third (34.7%) of the patients admitted to the acute psychiatric ward at CMJAH during the study period had abnormal neuro-imaging results, of which the vast majority were CT brain scans and the most commonly diagnosed pathology was atrophy (31.1%).

Similar yields have been reported by both local and international studies. Bennimahadeo et al.^20^ reviewed CT scans of 507 consecutive patients referred to Townhill Hospital in Pietermaritzburg, KwaZulu-Natal between 2013 and 2014. The authors reported that abnormality was recorded in 31% of scans and that radiological evidence of cerebral atrophy, infarcts, cysts and calcific foci was present in 30% of these patients. Chhagan et al.^4^ conducted a retrospective study in the psychiatry unit of Addington Hospital, Durban, KwaZulu-Natal, analysing all psychiatric inpatients who had CT scans performed between 2011 and 2012, and found that of a total of 897 admissions, 103 patients had documented CT scan imaging and 22% patients were abnormal, of which the majority had radiological evidence of cerebral atrophy and/or cerebral calcifications.

Jeenah et al.^21^ in their study of 600 admissions to Chris Hani Baragwanath Academic Hospital in Soweto, Johannesburg also reported that, of the 55 patients who underwent CT scans, 36.4% were abnormal. Jain et al.^22^ conducted their study in the Department of Radio-diagnosis at the TSM Medical College, Lucknow, Uttar Pradesh, India, on 95 psychiatric patients of which 47.3% had abnormal scans. Hollister et al.^23^ conducted a study, extending 3 years, at the Harris Country Psychiatric Center in Houston, Texas, which included neuro-imaging reports of 337 psychiatric patients and found that 35% of cases were abnormal.

However, some studies have reported a much higher yield. A recent study by Juby et al.^24^ at Townhill Hospital in Pietermaritzburg, KwaZulu-Natal, retrospectively reviewed the charts of all psychiatric patients who had neuro-imaging between 2010 and 2016 and found that of the 53 MRIs performed, 62% were abnormal. Gupta et al.^25^ studied 102 consecutive patients referred for CT brain scanning for primary psychiatric diagnosis in 2003 in the Department of Radiology at West Wales Hospital, Carmarthen. The indications for referral for CT brain were based on the clinical judgement of the psychiatrist and the results showed that 64% showed some abnormality. In a prospective study by Chandler et al.,^26^ of the 42 new geriatric psychiatry patients included, 37 underwent CT scanning, of which 86% were abnormal. The authors suggest that the high yield was most likely because these were geriatric patients and the main abnormal finding was that of atrophy.

In contrast, other studies have reported lower yields. LeBaron et al.^27^ reviewed the neuro-imaging reports of 224 patients admitted to Alberta Hospital, Edmonton, Canada, between 2012 and 2015. They reported that only 13.4% of scans were classified as abnormal, of which 10.7% were deemed benign and non-specific. Sommer et al.^28^ investigated the prevalence of clinically relevant abnormalities as part of a series of brain imaging studies at the Neuroscience Division of the University Medical Center, Utrecht between 1995 and 2010. Of the 656 psychotic patients who underwent MRI scans, they found clinically relevant pathology in only 11.1% of the patients. Agzarian et al.^29^ retrospectively assessed the reports of 397 consecutive CT brain scans of patients presenting to two acute tertiary hospital psychiatric services over 2 years and found that scan abnormalities were reported in only 5% of patients and were not related to the patients’ psychiatric condition. Hollister et al.^16^ studied all scans ordered in a psychiatric teaching hospital over approximately 2 years and reported that of the 68 scans reviewed, only 17% were abnormal. McClellan et al.^30^ conducted a retrospective audit of the CT brain scans of 261 patients admitted with psychiatric symptoms over 3 years and reported that only 12% of their scans were abnormal and the abnormalities consisted of cortical atrophy, basal ganglia calcifications and old lacunar infarction, all of which were unrelated to the patients’ psychiatric condition.

It is evident from the literature that the yield of abnormal neuro-imaging varies greatly and ranges from a low of 5% to a high of 86%. The wide variation in the different studies may be attributed to a variety of reasons, namely, different patient populations studied, inpatient versus outpatient, varying duration of illnesses, different age groups, variable criteria used for the eligibility of patients for CT scans by psychiatrists and differences in interpretation of scans by radiologists. It would appear that the more stringent the criteria, the lower the yield.

Computerised tomography scans are quick and sensitive imaging test for the majority of brain lesions^31^ and relatively cheaper than MRI, whose average cost may be up to R10 000.^32^ In South Africa, the average cost of a CT brain scan in 2016 was between R3000 and R5000.^33^ This expensive diagnostic procedure remains exclusively available at the tertiary hospitals and is not easily accessible in Gauteng. Often these scanners are not working well because of poor maintenance, resulting in long waiting times for the investigation to be done – sometimes up to 6 weeks for inpatients and 6 months for an outpatient.^33,34^ There are also problems associated with CT scans. The approximate effective radiation dose of a contrasted CT brain scan is 4 millisieverts, which is comparable to 200 chest X-rays.^33,35^ This radiation exposure and its risk must be weighed against the possible benefit of investigative CT brain scans.^31^ If used unselectively, it may result in the discovery of incidental findings that may have unimportant implications.^33^

Taking into consideration the limited accessibility of CT scans in Gauteng hospitals, and the low yield of abnormal results, the practice of routine brain scans screening for all first presentations to psychiatry needs to be reviewed. There is a wide divergence of opinion regarding this issue. The current American Psychiatric Association guidelines recommend brain imaging in FEP and favour either MRI or CT scans.^17^

However, some other national guidelines do not make similar recommendations.^36,37^

Although studies have reported abnormal CT scan findings in subjects with FEP,^38,39^ in a recent large retrospective cohort study, the authors reported little diagnostic value in CT scans in this group.^33,40^ They also reported that the rates of incidental abnormalities are similar to that observed in the healthy general population.^41^ Albon et al.^42^ suggested that if screening with structural neuro-imaging was implemented in all patients presenting with psychotic symptoms under 65 years old, little would be found to affect clinical management in addition to that suspected by a full clinical history and neurological examination. Advocating imaging scans as a screening procedure for all patients will not improve the low yield, whilst accepting the risk of missing a rare diagnosis.^25^ Routine neuro-imaging as a diagnostic workup for all first psychiatric presentations with normal neurological examination should be reviewed.

However, in patients with clear focal neurological abnormalities or clinical predictors of intracranial abnormalities, CT screening is indicated. Certain neurological disorders of the brain, which may be reversible, may present initially or solely with psychiatric signs and symptoms and acute altered mental statuses (22).^23^

The yield of abnormal scans is, therefore, likely to be increased if psychiatric patients have a comorbid neurological illness or abnormal neurological signs.^4,18,19,21,27,39^ This study found that approximately one out of four patients (24.4%) with abnormal scans was diagnosed with comorbid HIV infection. Neurological illness and signs in HIV may arise as a result of the virus directly causing neuronal death and atrophy,^39^ because of psychosocial stressors or because of complications of antiretroviral therapy.^39,41^

Mental illness may also increase an individual’s risk for HIV infection through increased social vulnerability, risky sexual relationships and comorbid substance use.^43^

The HIV prevalence rate of this study is similar to the rates amongst mental healthcare users reported in other South African studies. Singh et al.^44^ reported an HIV prevalence of 29.1% in an acute psychiatric ward in KwaZulu-Natal.^44^ Collins et al.^45^ also found a prevalence of 26.5% in a public psychiatric institution in KwaZulu-Natal.^45^

Mashaphu et al.^46^ in a subsequent study at Town Hill Hospital in KwaZulu-Natal reported a prevalence rate of 23.8%.^46^ In Gauteng, Van Rensburg et al.^47^ found a much higher prevalence of 44.2%.^47^ The findings in South Africa are also similar to the prevalence rates of 31% reported by Opondo et al.^48^ in Botswana, 23.8% by Acuda and Sebit^49^ in Zimbabwe and 18.4% by Maling et al.^50^; the prevalence ranged from 9% to 23%.^48,49,50^ The authors comment that the results should be interpreted with caution because the procedures for conducting HIV tests varied across the studies and most of the studies lacked comparative controls.

Computerised tomography scan is important in screening for radiological changes in HIV patients because of its non-complicated technique, relatively low cost and high speed of acquisition of images.^51^ The findings of CT scan can be divided into four categories: direct effect of HIV infection, opportunistic infections, vascular diseases and neoplasm.^52^ The findings include features of age-inappropriate brain atrophy, white matter disease (WMD), toxoplasmosis, cryptococcal, progressive multifocal leukoencephalopathy (PML), tuberculoma, tuberculous abscess, tuberculous meningitis, bacterial meningitis, cerebritis, lymphoma and stroke. Because there is such a high prevalence rate of comorbid HIV infection amongst persons with mental illness, one would expect a high yield of abnormal CT scans amongst this group of patients.^52^

This study found that of the psychiatric patients with a comorbid diagnosis of HIV, only 16.2% had abnormal scans. Although it was not statistically significant, there was a trend towards an association between abnormal scan and a diagnosis of HIV infection. However, there are several published studies that show significantly higher yields. Berwers^53^ conducted a retrospective study at Groote Schuur Hospital, Cape Town, South Africa, which included 65 HIV psychiatric patients who received a CT scan during 2013–2015. The author reported that of the patients 55.38% were abnormal, with atrophy as the most common radiological finding.^53^ Hongsakul^52^ investigated 195 CT scans and reported that HIV encephalopathy was the most common brain finding affecting 59% of the study population. Seth et al.^54^ reported that amongst HIV/AIDS patients with a psychiatric illness referred to liaison psychiatry in their study, a total of 23 CT scans were ordered and 73.9% were found abnormal. Robert^55^ found that 119 of the 200 CT scans (59.5%) in their study population of homosexual men were abnormal, with 75 showing atrophy and 44 showing a focal lesion. Grill et al.^56^ in their study of 305 MRIs performed on HIV-positive individuals over 15 years reported that 86% had abnormal scans. The authors further reported that subcortical white matter changes and cerebral atrophy were the common pathologies. Juby and Paruk^24^ reported that 83% of the HIV-infected subjects had abnormal MRIs.

As this study was a retrospective record review, it is possible that not every patient was subject to an HIV test, thus accounting for the low yield of abnormal scans. In a recent article, Juby et al.^24^ also observed that the HIV status in 22.6% of their patients was unknown. They attributed this to the problems associated with obtaining informed consent for HIV testing from some mental healthcare users.^24^ Joska et al.^57^ for the use of ‘clinician-initiated testing’ proposed by the Joint United Nations Programme on HIV/AIDS (UNAIDS) and the World Health Organization (WHO) recommended that in mental health care users who do not have the capacity to make this decision, a senior clinician and/or the medical superintendent should be consulted to weigh up the potential harms and benefits of HIV testing and approve testing.^57^

This study did not find any significant association with the psychiatric diagnosis on admission and having an abnormal scan as done in other similar studies.^5,8,35^ This supports the premise that the actual role of structural neuro-imaging in psychiatry remains unclear. Currently, it serves only to strengthen available clinical evidence in confirming a psychiatric diagnosis.^4,5^ The study also did not find any significant association between substance use and having an abnormal scan which was similar to the findings of Chhagan et al.^4^

The majority of abnormal scans in this study were in 30–39 years age group. This is unusual as the majority of researchers report a correlation between advancing age and abnormal CT scans.^4,20,23,28,49^ It is possible that because of the high incidence of HIV amongst our patients, the peak age for abnormal scans was much lower. Based on point estimates, the overall HIV peak prevalence reported occurs in 35–39 years age group.^58^

## Limitations

The convenience sampling method used in this study to select all of the consecutive cases within a given time frame may have resulted in a selection bias. The sampling method excluded patients who had neuro-imaging within the selected study period but no report limited the sample size, which may have distorted the measure of association. There was also a group of patients who were admitted from the emergency department of CMJAH directly to the ‘overflow’ ward to await a bed in the psychiatric ward. Some of these patients may have had neuro-imaging tests performed and discharged without reaching the psychiatric ward and consequently were not included in the study.

Typically with retrospective studies, partial and missing information in medical records led to non-response bias in the results. The neuro-images were reported by medical practitioners with varying skills. Although they may have been validated by a specialist radiologist, the reporting of the pathology on the neuro-images had the potential to introduce information bias.

## Recommendations

At the time of this study, the practice at the CMJAH Radiology Department was to conduct neuro-imaging as follows:

1. As a routine screening for all first presentations to psychiatry.
2. When there were medical indications.
3. Emergency scans are done on the same day and others ‘as soon as possible’ – within 2 weeks of the request.
4. The first neuro-imaging investigation is a CT brain scan. An MRI is only done when the CT brain scan does not provide sufficient information and/or when more clinical information is required for a diagnosis.

It is recommended that further research should be conducted to assess the clinical factors that are associated with abnormal neuro-imaging results in psychiatric patients, their role in diagnoses, monitoring disease progression and predicting prognoses of psychiatric disorders and the cost-effectiveness of this intervention in constrained settings.

In the interim, neuro-imaging should not be done as routine screening for the first presentation to psychiatry, but rather for those patients who have focal neurological signs following a full neurological examination and/or those who are positive following an HIV test. It is appropriate to first do a CT scan, followed up by an MRI if radiologically indicated. Dougherty et al.^59^ recommend the use of MRI for ruling out white matter lesions, infarcts, contusion, infection and new-onset psychiatric symptoms in subacute presentations.

## Conclusion

This study supports the need for clear clinical indications in resource-constrained settings, such as South Africa, to justify the cost-effective use of neuro-imaging in psychiatry. This study’s relatively high yield of abnormal CT scans, although similar to other studies, advocates that HIV positive testing and the presence of focal neurological signs will improve the yield further.
